# Comparison of Artificial Anterior Chamber Internal Pressures and Cutting Systems for Descemet's Stripping Automated Endothelial Keratoplasty

**DOI:** 10.1167/tvst.7.6.11

**Published:** 2018-11-27

**Authors:** Sota Nishisako, Dai Aoki, Chiaki Sasaki, Kazunari Higa, Jun Shimazaki

**Affiliations:** 1 Cornea Center and Eye Bank, Tokyo Dental College, Ichikawa General Hospital, Chiba, Japan

**Keywords:** Descemet's stripping automated endothelial keratoplasty, microkeratome, artificial anterior chamber internal pressure, donor tissue quality parameters

## Abstract

**Purpose:**

To optimize methods of preparing donor cornea tissue for Descemet's stripping automated endothelial keratoplasty (DSAEK), we compared five experimental conditions with different internal pressures and cutting systems.

**Methods:**

The artificial anterior chamber internal pressure (IP) was set at 100 or 200 mm Hg. The microkeratome cut was performed with or without an artificial chamber pressurizer (ACP), using a CBm turbine (CBm) or one use-plus automated (OUP-A). Thirty human research corneas were divided into five groups, and compared after the cut with donor tissue quality parameters, including cutting depth, graft uniformity, cell evaluation, and smoothness of the stromal surface.

**Results:**

The smallest variation in mean cut depth was observed in the condition, which had IP of 200 mm Hg used ACP and OUP-A. In experimental groups cut using CBm, significantly more consistent thicknesses were made at an IP of 200 than 100 mm Hg. There were no statistically significant differences among the groups in either endothelial cell density or cell viable assay results after cuts. Using an IP of 200 mm Hg with ACP and CBm produced the roughest stromal surface, and the roughness grading scores showed a positive correlation with the percentage of cut depth.

**Conclusions:**

An IP of 200 mm Hg was the best setting for DSAEK grafts with high predictability of cut depth and uniformity of graft thickness without endothelial cell damage.

**Translational Relevance:**

For successful DSAEK, it is recommended that a set internal pressure of 200 mm Hg be used during microkeratome cutting for donor tissue preparation.

## Introduction

Descemet's stripping automated endothelial keratoplasty (DSAEK) is currently the most widely used procedure for treating corneal endothelial dysfunction, such as Fuchs' endothelial dystrophy and pseudophakic bullous keratopathy.^1^ DSAEK allows rapid visual recovery and minimum astigmatism compared with penetrating keratoplasty.^1–3^ Despite its several advantages, its resultant postoperative best-corrected visual acuity has been reported to be between 20/40 and 20/30.^4,5^ While many factors influence visual outcomes after DSEAK, one of the most common reasons for poor visual recovery is caused by irregular donor graft thicknesses.^6–8^

DSAEK tissue is most often processed by a microkeratome.^9^ The microkeratome cutting depth is adjusted to control the thickness of the resulting posterior lenticule.^10^ Even if the same-sized head is used for all cutting, it produces a wide range of final tissue thicknesses, because the relationship between microkeratome head size and final tissue thickness is imprecise.^11,12^ Therefore, some studies have shown that DSAEK grafts prepared using a microkeratome produce nonuniform thickness profiles and variable central graft thickness.^8,11–14^ Previous studies have evaluated the quality of DSAEK lenticles using various methods, including endothelial cell counting,^15^ thickness measurements,^16,17^ and smoothness of surface measurements.^18,19^ Although it is essential to achieve high reproducibility and uniformity in graft thickness with minimal tissue damage when preparing donor tissue for successful DSAEK, the optimum cutting method to produce a DSAEK graft has not been established. Importantly, maintenance of internal pressure (IP) during microkeratome cutting plays a crucial role in producing a reproducible cutting profile.

Recently, artificial chamber pressurizer (ACP) and one use-plus automated (OUP-A) are getting a lot of attention as new pressurization method and new cutting system. ACP maintains a constant IP during the microkeratome cut with a preset level. OUP-A is a self-running microkeratome, with a pass that is automatically performed by the instrument. These new devices are expected to produce high-quality DSAEK grafts.

In this study, we optimized the IP and cutting method for human donor corneal grafts. Our goal was to develop a methodology to provide high-quality DSAEK grafts with high reproducibility of cut depth, uniformity of graft thickness, and smoothness of the cutting-stromal surface without endothelial cell damage. We compared five experimental conditions with different artificial anterior chamber IPs and cutting systems using the new devices.

## Materials and Methods

### Human Corneas

Thirty human corneas with consent for use in research were obtained from a US eye bank (SightLife, Seattle, WA). Corneoscleral specimens were preserved in a viewing chamber (Krolman, Boston, MA) and kept in corneal storage medium (Optisol-GS solution; Bausch & Lomb Surgical, Rochester, NY). All donor tissues were shipped internationally by airplane at 4°C. Donor ages, donor sex, and death to preservation time were all recorded.

### Experimental Groups

All donor tissues were warmed to room temperature before the microkeratome cut. Each corneoscleral specimen was mounted on an artificial anterior chamber (Moria, Antony, France) and stamped using a sterile skin marker (Koken, Tokyo, Japan) to mark the entry point of the cut. The initial IP was set at 100 or 200 mm Hg. For pressure control (PC), a conventional method using an infusion bottle (conventional group; IP is unstable during the cut), and a new method using ACP (IP is stable, Moria) were compared. The IP of conventional group was adjusted by inflating a pressure cuff surrounding the infusion bottle that was set at 4-ft height from working area surface. The tube clamp was closed to increase the IP to 100 or 200 mm Hg. Similarly, the dial of the APC was set to 100 or 200 mm Hg. The initial IP was measured with a real-time pressure sensor (nVision Reference Recorder; AMETEK, Berwyn, PA) connected to the infusion line leading to an artificial anterior chamber. The IP changes during the microkeratome pass was recorded every 1/10 second. Two types of microkeratomes, CBm turbine (CBm; Moria) with manual rotative cutting and OUP-A (Moria) with automatic linear cutting, were compared. The cutting was performed by the same technician with a 350-μm head and a new blade for each specimen. The cutting speed was approximately 3 seconds for CBm or was 3.0 mm/s by selecting at “speed 2” on the Evolution 3E control unit (Moria) for OUP-A. The separated anterior lamella cap was replaced onto the cornea and returned to the viewing chamber filled with the corneal storage medium. Thirty human research corneas were divided into five groups (groups A–E) with various IPs, PCs, and two types of microkeratome (CBm/OUP-A) settings as follows: (A) IP, 100 mm Hg; PC, conventional (infusion bottle); Microkeratome, CBm (manual); (B) IP, 100 mm Hg; PC, ACP; Microkeratome, CBm; (C) IP, 200 mm Hg; PC, conventional; Microkeratome, CBm; (D) IP, 200 mm Hg; PC, ACP; Microkeratome, CBm; and (E) IP, 200 mm Hg; PC, ACP; Microkeratome, OUP-A (automatic; Table 2).

### OCT Scanning of Corneas

Corneal thickness before and after the microkeratome cut was measured using anterior-segment optical coherence tomography (OCT; CASIA SS-1000; TOMEY, Nagoya, Japan). The scan rate of the Fourier-domain OCT (CASIA SS-1000) was 30,000 axial scans per second with a 10-μm axial resolution in tissue. Each corneoscleral specimen was scanned through the transparent window of the corneal viewing chamber without taking them out of the storage medium. The chamber was held on the chinrest by a custom-built attachment with the mounted cornea facing the image capture lens with the entry point of the cut in the bottom to maintain a standardized entry of the upward cut. Images were obtained along two meridians (horizontal and vertical to the microkeratome pass line). In each scan line, the thickness of the corneoscleral disc was measured at the central zone and at 3 mm from the center in manual mode. The cut depth, calculated by subtracting the thickness before and after the cut at the center, was compared among groups. The thickness of the peripheral was measured at entry, exit, left, and right at 3 mm from the center of the microkeratome pass line on the graft. The mean differences between the maximum and minimum of marginal regions were compared as graft thickness uniformity.

### Endothelial Cell Damage

The central endothelial cell density (ECD) was measured in all corneal specimens using a specular microscopy (EKA-10 KeratoAnalyzer; Konan Medical, Hyogo, Japan) before and after the cut. Ten corneas (2 from each experimental group chosen at random) were stained and used for endothelial cell viability assays. The sectioned corneas, stored at 4°C for 2 days, were warmed to room temperature and then rinsed in phosphate-buffered saline (PBS) and punched with 8-mm trephine (Kai Medical Industries, Gifu, Japan). The trephined grafts were placed endothelial side up in 6-well sterile Petri dishes in the dark for 5 minutes with 2-mL 1 μM Hoechst 33342 (Dojindo Laboratories, Tokyo, Japan) in a 1:1 (vol*/*vol) mixture of Dulbecco's modified Eagle's medium (Life Technologies, Carlsbad, CA) and Ham's F12 (Life Technologies). After adding 10-μM propidium iodide (Wako Pure Chemical Industries, Osaka, Japan), the specimens were gently rinsed in PBS and transferred to a glass slide. Images were taken using a fluorescence microscope (Axioplan 2; Carl Zeiss, Thornwood, NY) at three different central locations. All positive Hoechst 33342/propidium iodide nuclei were counted using Image J software (National Institutes of Health, Bethesda, MD). The mortality rate was calculated as the number of nuclei that stained positive with propidium iodide out of the total number of nuclei that stained positive with Hoechst 33342.

### Scanning Electron Microscopy

Two corneas were chosen from each of the five experimental groups, and they were fixed in 2.5% glutaraldehyde (TAAB Laboratory Equipment, Berks, UK) in 60-mM HEPES buffer (pH 7.4) at 4°C. After fixation, they were postfixed in buffered 2% osmium tetroxide (Heraeus Chemicals, Port Elizabeth, South Africa) for 2 hours in an ice bath. Then they were dehydrated in a graded ethanol series (30%–100%; Nacalai Tesque, Kyoto, Japan). After several changes in 100% ethanol, the specimens were dried using a t-butyl alcohol (Kanto Chemical, Tokyo, Japan) freeze-drying method. The dried specimens were mounted on aluminum stubs and coated by an osmium plasma ion coater (OPC-80; Nippon Laser and Electronics Laboratory, Nagoya, Japan). Images were acquired using a scanning electron microscope (SEM; JSM-6320F; JEOL, Tokyo, Japan) at 5 kV. The samples were observed at ×20 and ×200 magnification. The observed areas were the total microkeratome-cut stromal cap surface (×20) and three central points (×200). Whole images of specimens were obtained by connecting ×20 magnification SEM images.

### Stromal Surface Roughness Grading

Qualitative surface roughness values of the five experimental groups were compared using the methodology described by Sarayba et al.^18^ All SEM images of microkeratome-cut stromal cap surfaces were graded from 1 to 5 (1 = smoothest, 2 = next smoothest, 3 = median, 4 = rough, and 5 = roughest) by three masked observers. The scores for individual specimens were averaged and compared between groups.

### Statistical Analyses

The data were tabulated and compared between experimental groups using Prism software, version 6.04 for Windows (GraphPad Software, La Jolla, CA). The normality of the data was assessed using Kolmogorov-Smirnov tests. Bartlett's test and the F-test were used to assess whether the data showed equal variance. The cut depth and graft thickness were used to compare the IP and the PC of multiple groups using two-way analysis of variance, and also to compare the microkeratome differences via Student's *t*-tests. Data on endothelial cell damage and qualitative surface roughness were compared between groups using Kruskal-Wallis tests with the Dunn's multiple comparisons test and the Mann-Whitney *U* test. A value of *P* < 0.05 was considered statistically significant.

## Results

### Baseline Characteristics of Donor Corneas

There were no statistically significant differences among the five groups regarding donor age, sex, death to preservation time, central cornea thickness (Table 1), and ECD before cutting (Table 3).

**Table 1:**
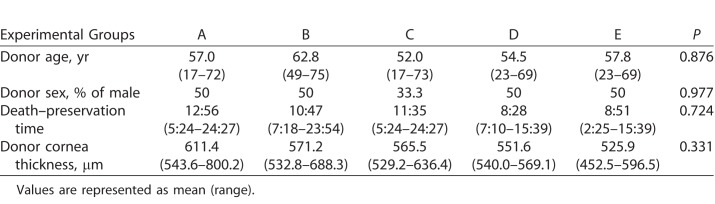
Donor Demographics

**Table 2:**
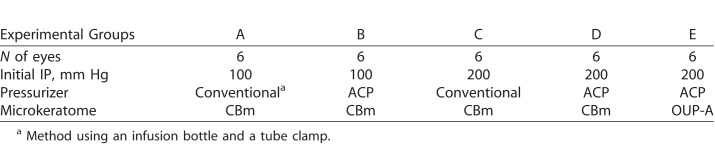
Experimental Groups

**Table 3:**
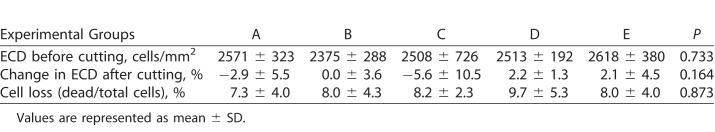
Change in Endothelial Cell Density (ECD) and Cell Loss After the Cut

### Changes in IP During the Microkeratome Pass

**Figure 1:**
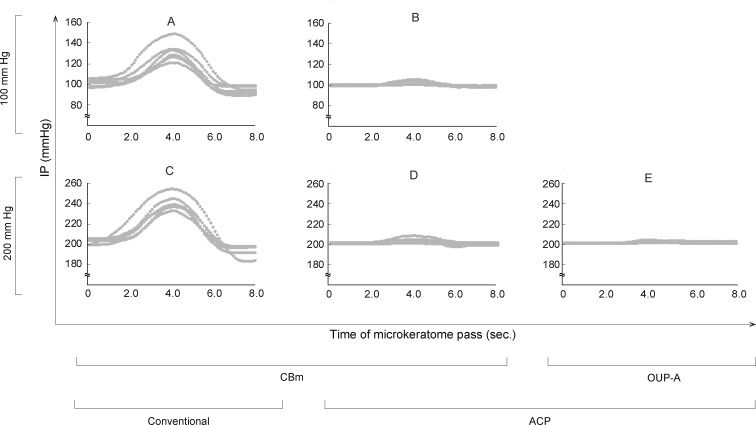
Changes in artificial anterior chamber IP at microkeratome passing. The IP change during dissection was measured every 1/10 second by a real-time pressure sensor connected to the infusion line. While the groups in which ACP was used (groups B, D, and E) showed constant IPs, the conventional groups (groups A and C) had elevated IPs during the microkeratome pass.

The results of the IP changes are shown in Figure 1. While the groups in which ACP was used (groups B, D, and E) showed constant IPs, the conventional groups (groups A and C) had elevated IPs during the microkeratome pass, followed by a decrease at the initial set point. Moreover, the peak levels were variable for each cut. The ranges of the IPs for each group were as follows: (A) 89.7–147.1 mm Hg (mean 109.2 ± 12.3 [SD] mm Hg); (B) 97.8–105.2 mm Hg (100.2 ± 1.1 mm Hg); (C) 177.7–258.1 mm Hg (213.2 ± 18.4 mm Hg); (D) 195.0–207.8 mm Hg (199.9 ± 1.6 mm Hg); and (E) 198.8–202.9 mm Hg (200.4 ± 0.7 mm Hg).

### Corneal Thickness Profiles

**Figure 2:**
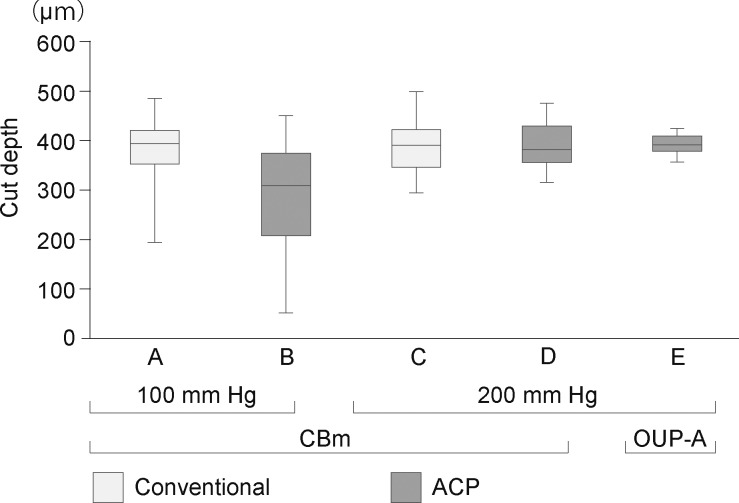
The cut depth of each of the five groups measured using anterior-segment OCT. Error bars represent standard deviations. There were no statistically significant differences among the groups. Variation in cut depth tended to be smaller in the IP 200-mm Hg group than the IP 100-mm Hg group.

**Figure 3:**
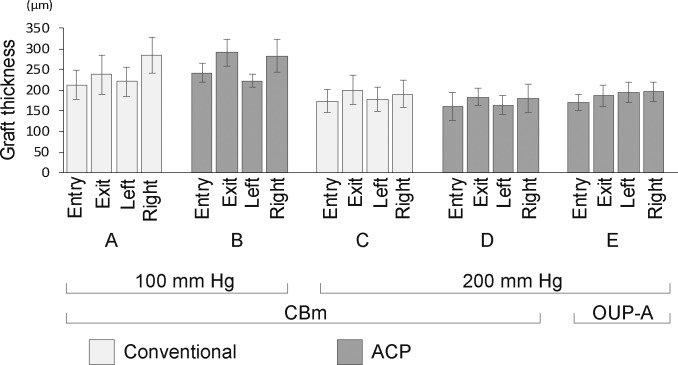
The mean residual graft thickness profiles of marginal regions at 3 mm from the center (entry, exit, left, and right for the microkeratome pass line) after the cut. Error bars represent standard deviations. There were tended to be thinner at the entry side than the exit side. In a similar manner, the left side was thinner than the right side at the microkeratome pass line.

**Figure 4:**
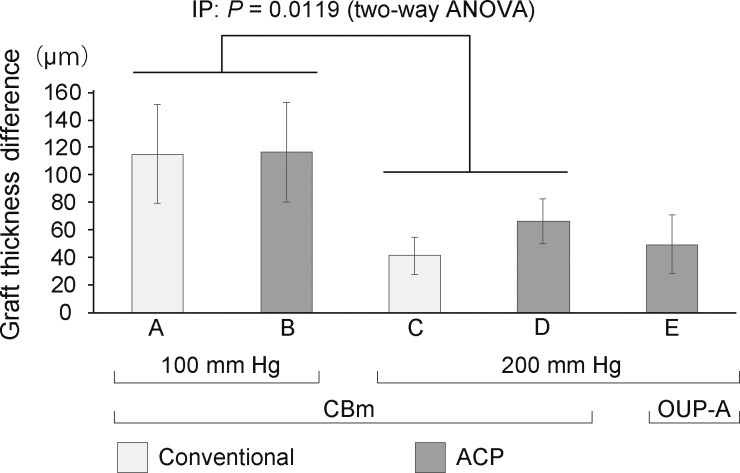
Comparison of peripheral residual graft thickness uniformity after the cut. Error bars represent standard deviations. It was significantly more consistent in groups cut by the CBm at IP 200 mm Hg than at IP 100 mm Hg.

The cut depth and mean anterior cap thickness at the center of each specimen did not differ among the groups (Fig. 2). The ranges of the cut depths were as follows: (A) 197.6 to 485.2 μm (375.4 ± 98.9 μm); (B) 56.5 to 451.8 μm (284.5 ± 145.5 μm); (C) 298.4 to 500.0 μm (393.3 ± 70.8 μm); (D) 318.6 to 477.3 μm (393.4 ± 58.4 μm); and (E) 361.1 to 427.3 μm (395.5 ± 24.6 μm). Variation in cut depth tended to be smaller in the IP 200- than the IP 100-mm Hg group. The smallest CV was obtained in group E, in which an IP of 200 mm Hg was applied using ACP and OUP-A. The post-cut graft thickness profiles at 3 mm from the center were similar among the groups, and tended to be thinner at the entry side than the exit side. In a similar manner, the left side was thinner than the right side at the microkeratome pass line (Fig. 3). Residual graft thickness uniformity in marginal areas was more consistent in groups cut by the CBm at IP 200 mm Hg than at IP 100 mm Hg (*P =* 0.0119; Fig. 4). The mean differences between the maximum and minimum thicknesses ranged as follows: (A) 34.6 to 229.7 μm (115.2 ± 72.0 μm); (B) 32.4 to 219.6 μm (116.5 ± 72.0 μm); (C) 5.8 to 70.6 μm (41.3 ± 27.2 μm); (D) 36.7 to 122.4 μm (65.9 ± 32.4 μm); and (E) 5.8 to 117.4 μm (49.4 ± 42.8 μm). There were no statistically significant differences in graft thickness between the PCs and the microkeratome types.

### Endothelial Cell Damage

The changes in ECD before and after the cut were from −5.6 to +2.2%, and there were no statistically significant differences among the five groups. The mortality ratio of endothelial cells was less than 10% in all groups, and post-cut cell viability assays indicated no significant differences among groups (Table 3). No serious endothelial cell damage was observed in any group.

### Observation of the Microkeratome-Cut Surface

**Figure 5:**
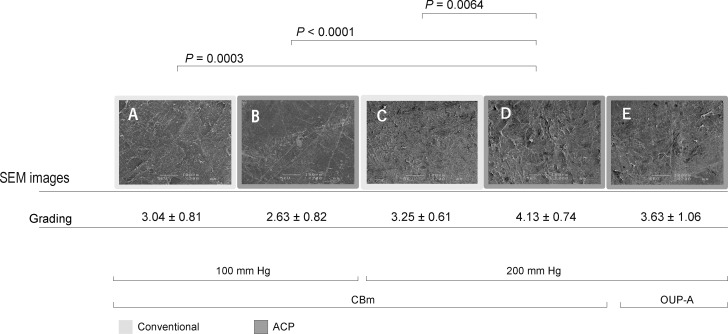
SEM images (×200) of the microkeratome-cut stromal cap surface and qualitative surface roughness grading of the five experimental groups. Group D, in which CBm was used at an IP of 200 mm Hg, had the roughest surface in SEM roughness grading.

Group D, in which CBm was used at an IP of 200 mm Hg, had the roughest surface in SEM roughness grading. Conversely, the smoothest cuts were observed for group B, in which CBm was used at an IP of 100 mm Hg (Fig. 5). There were statistically significant differences between group D and A (*P =* 0.0003), D and B (*P* < 0.0001), and D and C (*P =* 0.0064).

## Discussion

We optimized the cutting method for donor graft preparation for DSAEK. We examined five experimental conditions with different IPs and cutting systems (Table 2).

Control of the IP in microkeratome cutting has not been standardized, and various methods have been used including adjusting hydrostatic pressure by elevating a balanced salt solution bottle,^10,11,19^ inflating with air from a syringe,^2,3,7,8,19^ and ocular tonometry measuring a tonometer.^13,20,21^ We found that IP control using the conventional method produced high variation in IP level during cutting, and the peak IP levels varied among cuts (Figs. 1A and 1C). The maintenance of constant IP is important, as our data showed that variation in the thickness and uniformity of grafts depended on IP. Specifically, using an IP of 200 mm Hg with the ACP and OUP-A devices produced the smallest variation in cut depth (Fig. 2). In groups cut using the CBm device, applying an IP of 200 mm Hg led to significantly more consistent graft thickness uniformity than applying an IP of 100 mm Hg (Fig. 4). These results suggest that an IP of 200 mm Hg leads to more predictable cut depths and thickness uniformities. The high IP probably produced constant surface contours during microkeratome cuts. Previous studies have reported that the thickness of DSAEK grafts influences postoperative visual acuity,^4,6,14^ and visual recovery is faster in eyes with thinner and more uniform grafts.^21,22^ Thus, we suggest that graft preparation using a 200-mm Hg IP produces better visual recovery after DSAEK. This system may help with the preparation of a so-called “ultrathin DSAEK” graft with high predictability of cutting depth with reduced risk of perforation.^21,23,24^ We found that the mean percentage of endothelial cell loss preserved for 2 days after the cut was less than 10% in all groups, and there were no statistically significant differences among the five conditions. This is similar to rates reported in previous studies.^15,19^ Vito et al.^25^ reported that they performed DSEAK surgery using grafts prepared under IP of 198.8-mm Hg condition for 10 patients whom their corneas were clear 10 to 14 weeks after surgery. Altogether, this might indicate that cuts using a high IP did not cause significant damage of the corneal endothelial cell viability or its function.

It should be noted that there was a positive correlation (*r* = 0.667, *P =* 0.040) between the percentage of the cut depth for the overall corneal thickness (54%–89%) and roughness grading scores of each specimen (2.33–4.42) in all groups. Experimental group D, in which an IP of 200 mm Hg was used with the ACP and CBm devices, showed the roughest cutting surface. The corneal stroma is composed of more than 250 stacked collagen lamellae, each being 0.2- to 2.5-μm thick and forming domains (0.5- to 250-μm width) composed of collagen fibrins (25- to 35-nm diameter).^26^ Studies using second harmonic generation microscopy have shown that posterior stroma with coarse-grained structures are composed of thick and wide lamellae.^27,28^ Based on these observations, it is possible that the roughest qualitative score of the cutting surface in group D may have resulted from a deep cut (mean percentage of the cut depth in group A, 68%; B, 58%; C, 77%; D, 77%; E, 72%).

The thicknesses of peripheral grafts showed a similar tendency regardless of different cutting conditions (Fig. 3). Bhogal et al.^20^ also found that significant asymmetry was observed in donor lenticule cutting using a CBm microkeratome with a deep cut and thin lenticule at the beginning of the microkeratome pass, regardless of cutting speed. Furthermore, because the anterior and posterior donor corneal surface are not parallel, the grafts prepared by microkeratome might show some asymmetry to the final graft thickness in peripheral areas of the graft relative to the center of the graft.^6,7,8,14^ This indicates that graft thickness was nonuniform even when cutting conditions were controlled. Our data will be valuable for the future development of donor preparations with uniform thicknesses, but further studies are required to improve uniformity in peripheral graft thickness.

In conclusion, an IP of 200 mm Hg was the best setting for donor tissue preparation for DSAEK grafts, resulting in high predictability of cut depth and uniformity of graft thickness without endothelial cell damage. ACP was useful for setting a constant IP of 200 mm Hg. Although further studies are needed, the OUP-A provided high reproducibility of cutting thickness.
